# Cable Pili and the Associated 22 Kda Adhesin Contribute to *Burkholderia Cenocepacia* Persistence In Vivo

**DOI:** 10.1371/journal.pone.0022435

**Published:** 2011-07-21

**Authors:** Joanna B. Goldberg, Shyamala Ganesan, Adam T. Comstock, Ying Zhao, Uma S. Sajjan

**Affiliations:** 1 Department of Microbiology, University of Virginia Health System, Charlottesville, Virginia, United States of America; 2 Department of Pediatrics and Communicable Diseases, University of Michigan, Ann Arbor, Michigan, United States of America; Tulane University, United States of America

## Abstract

**Background:**

Infection by *Burkholderia cenocepacia* in cystic fibrosis (CF) patients is associated with poor clinical prognosis. Previously, we demonstrated that one of the highly transmissible strains, BC7, expresses cable pili and the associated 22 kDa adhesin, both of which contribute to BC7 binding to airway epithelial cells. However, the contribution of these factors to induce inflammation and bacterial persistence in vivo is not known.

**Methodology/Principal Findings:**

Wild-type BC7 stimulated higher IL-8 responses than the BC7 *cbl* and BC7 *adhA* mutants in both CF and normal bronchial epithelial cells. To determine the role of cable pili and the associated adhesin, we characterized a mouse model of *B. cenocepacia*, where BC7 are suspended in *Pseudomonas aeruginosa* alginate. C57BL/6 mice were infected intratracheally with wild-type BC7 suspended in either alginate or PBS and were monitored for lung bacterial load and inflammation. Mice infected with BC7 suspended in PBS completely cleared the bacteria by 3 days and resolved the inflammation. In contrast, mice infected with BC7 suspended in alginate showed persistence of bacteria and moderate lung inflammation up to 5 days post-infection. Using this model, mice infected with the BC7 *cbl* and BC7 *adhA* mutants showed lower bacterial loads and mild inflammation compared to mice infected with wild-type BC7. Complementation of the BC7 *cblS* mutation in *trans* restored the capacity of this strain to persist in vivo. Immunolocalization of bacteria revealed wild-type BC7 in both airway lumen and alveoli, while the BC7 *cbl* and BC7 *adhA* mutants were found mainly in airway lumen and peribronchiolar region.

**Conclusions and Significance:**

*B. cenocepacia* suspended in alginate can be used to determine the capacity of bacteria to persist and cause lung inflammation in normal mice. Both cable pili and adhesin contribute to BC7-stimulated IL-8 response in vitro, and BC7 persistence and resultant inflammation in vivo.

## Introduction

*Burkholderia cenocepacia* is an important opportunistic pathogen causing respiratory infections in individuals with cystic fibrosis (CF). It is a member of the *Burkholderia cepacia* complex (Bcc). The Bcc represents at least 17 phylogenetically closely related yet distinct species of bacteria that are commonly found in the environment and can serve as agents for both plant and human infection [1], [2], [3]. Although most Bcc species have been isolated from CF lungs, the two most common are *B. multivorans* and *B. cenocepacia*. Infections with certain strains of *B. cenocepacia* (in particular, those of the ET12 lineage) are associated with a variable and unpredictable clinical course ranging from asymptomatic carriage to a rapid decline in clinical condition leading to fatal necrotizing pneumonia and septicemia, also known as ‘cepacia syndrome’ [4].

In our earlier studies, we showed that ET12 strains that cause ‘cepacia syndrome’ bind to human respiratory mucins via a pilin-associated 22 kDa adhesin protein [5], [6]. This protein is distributed along the shaft of the large, peritrichous appendages known as cable pili [6]. We also showed that the 22 kDa adhesin mediates the adherence of cable-piliated *B. cenocepacia* to cytokeratin 13 (CK13), the expression of which is enriched in airway epithelial cells differentiated into the squamous phenotype [7], [8]. CK13 expression is also increased in CF airway epithelial cells, particularly in bronchiolar and respiratory epithelium [9]. This increased CK13 expression is not directly linked to mutation in the CF transmembrane conductance regulator (CFTR), but rather is due to repeated injury of the airway epithelium as observed in the lungs CF patients that can lead to squamous differentiation [10]. Therefore, it is conceivable that *B. cenocepacia* capable of binding to CK13 may have a greater potential to cause infection, particularly in CF. Consistent with this, we observed that *B. cenocepacia* strains that express both cable pili and the 22 kDa adhesin bind better to lung sections from CF patients compared to lung sections from normal individuals. Cable pili and 22 kDa adhesin expressing bacteria also showed increased binding to lung sections from CFTR knockout mice compared to sections from wild-type mice [9]. We showed that isogenic mutants of the ET12 lineage strain BC7 lacking either the cable pilus (BC7 *cblA* or BC7 *cblS*) or the 22 kDa adhesin (BC7 *adhA*) were attenuated in binding to and transmigration across squamous differentiated primary airway epithelial cells [11], suggesting that cable pili and the adhesin may be required for causing persistent infection *in vivo.*

Recently, we and others have shown that the suspension of bacteria in *Pseudomonas aeruginosa* alginate facilitates persistence of bacteria in both normal and CFTR knockout mice by delaying the initial innate immune responses required for bacterial clearance [12], [13], [14], [15]. Here we have further characterized *B. cenocepacia* infection model in normal mice and determined the capacity of BC7 cable pili mutants: BC7 *cblA,* BC7 *cblS,* and BC7 *cblS* mutant complemented with *cblS* in *trans*, and the BC7 *adhA* mutant to persist and cause inflammation *in vivo*. We also determined the capacity of these strains to stimulate IL-8 responses in airway epithelial cells.

## Results and Discussion

### BC7 *cblA*, BC7 *cblS*, and the BC7 *adhA* mutant show decreased stimulation of an IL-8 response in airway epithelial cells

**Figure 1:**
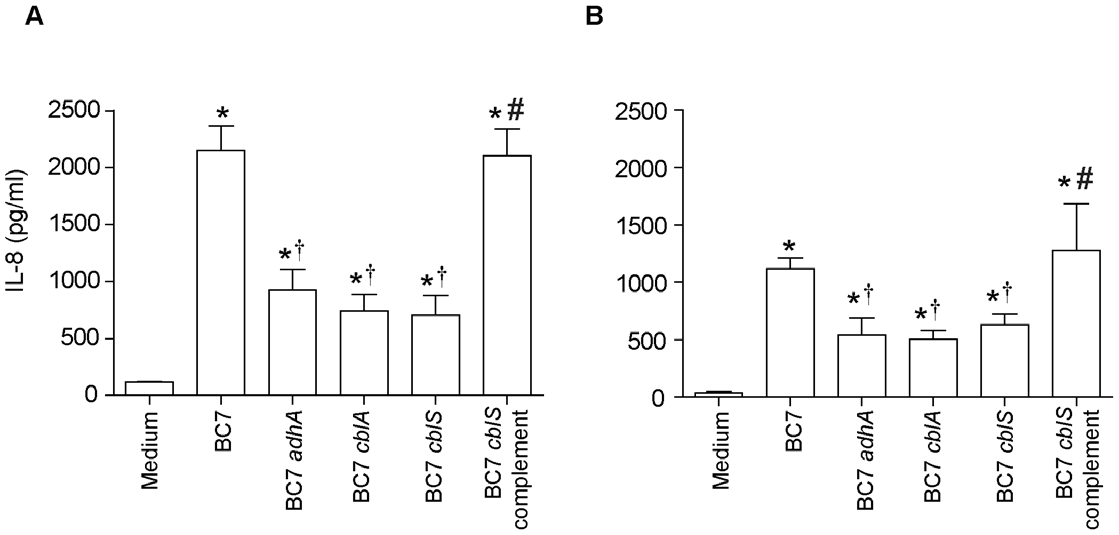
Stimulation of IL-8 response by *B. cenocepacia* strains in airway epithelial cells. IB3 (A) or BEAS2B (B) cells were treated with media or media containing BC7, BC7 *adhA*, BC7 *cblA*, BC7 *cblS*, or the BC7 *cblS* complemented strain (BC7 *cblS* complement), and IL-8 was determined by ELISA. Data represents mean ± SEM calculated from three independent experiments carried out in triplicates. (*different from medium control, † different from BC7, #different from BC7 mutants p≤0.05, ANOVA).

To assess the pro-inflammatory potential of bacteria, we infected IB3 (CF airway) epithelial cells with wild-type BC7, or the BC7 cable pili mutants (BC7 *cblA* or BC7 *cblS*) or the BC7 *adhA* mutant [11] and determined the IL-8 levels (Figure 1A). All strains showed significantly increased IL-8 production in CF cells compared to cells receiving only media. All three mutants stimulated approximately 2.5–3 fold less IL-8, compared to the wild-type BC7 strain.

To examine whether BC7 *cblA,* BC7 *cblS*, and BC7 *adhA* mutants were similarly attenuated in stimulating IL-8 response in normal airway epithelial cells, BEAS2B cells were infected with wild-type BC7 or the mutants, and IL-8 response was determined. Wild-type BC7 stimulated higher IL-8 production than all three BC7 mutants, although the absolute IL-8 levels were much lower than those observed in the CF airway epithelial cells (Figure 1B) and this may be due to decreased expression of CK13 and TNF receptor I in normal cells compared to CF cells [7], [9].

Previously, we demonstrated that 22 kDa adhesin is associated with cable pili and is required for binding to CK13 in both squamous differentiated cells as well as in undifferentiated normal airway epithelial cells, but the role of this interaction in stimulating IL-8 responses in airway epithelial cells was not investigated [8], [16]. Following this, we demonstrated that interaction of BC7 with TNF receptor 1 partly contributes to BC7-induced IL-8 and this phenomenon was not dependent on the expression of 22 kDa adhesin [7]. Here, using isogenic mutants of cable pili and the adhesin protein, we provide evidence that both cable pili and the 22 kDa adhesin in addition to facilitating binding to CK13, also play a role in BC7 stimulated IL-8 response in airway epithelial cells. These results suggest that BC7-stimulated IL-8 requires interaction of bacteria with both CK13 and TNF receptor I.

Attempts to complement the BC7 *cblA* and BC7 *adhA* mutants have been unsuccessful. Similar to our experience, Tomich et al [17] was also unable to complement a *cblA* mutant. Therefore we compared the BC7 *cblS* mutant and the mutant complemented with *cblS* in *trans* to show that the observed effects are specific at least for cable pili. BC7 *cblS* is mutated in the gene, *cblS*, which regulates expression of the *cblA* gene. BC7 *cblS* does not express cable pilus similar to the BC7 *cblA* mutant [11], [18]. The BC7 *cblS* complemented strain which expresses cable pili [11] stimulated IL-8 response very similar to wild-type BC7, indicating that cable pili is required for this trait. Together, these results suggest both cable pili and the associated 22 kDa adhesin also contributes to BC7 stimulated IL-8 in CF and normal airway epithelial cells.

### Characterization of *B. cenocepacia* infection model in C57BL/6 mice

**Figure 2:**
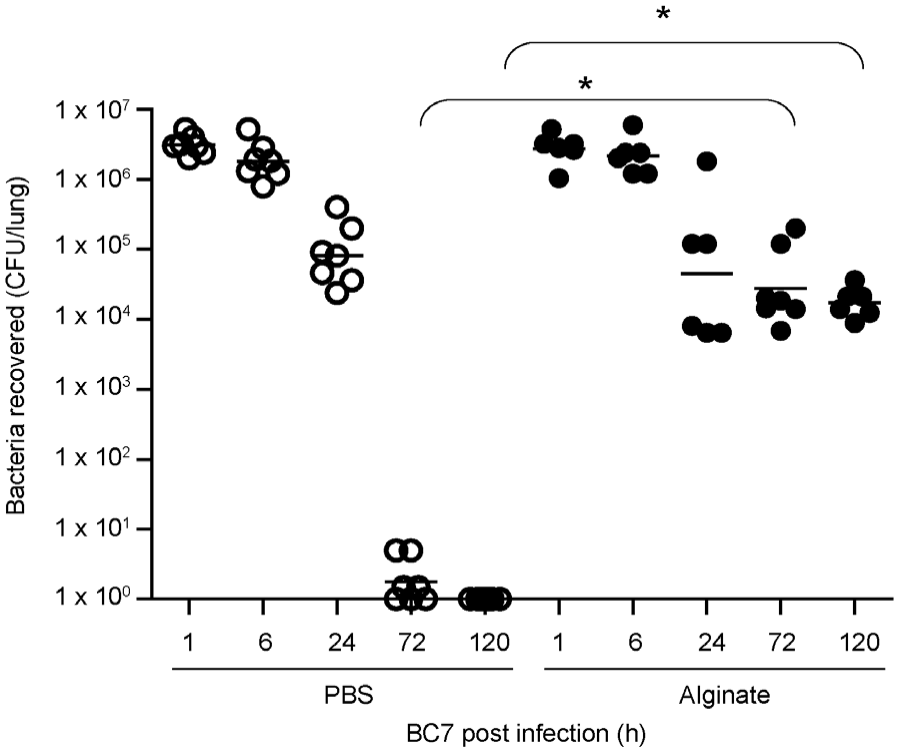
Lung bacterial load in C57BL/6 mice infected with BC7/PBS or BC7/alginate. Mice infected with BC7/PBS or BC7/alginate were sacrificed at pre-determined times, and ten-fold serial dilutions of lung homogenates were plated on BCSA to determine bacterial load. Data points represent individual mice and bars represent geometric mean calculated from three independent experiments with a total of 6 - 7 mice per group (*different from BC7/PBS group at respective time points, p≤0.05, ANOVA on Ranks).

Next, we tested whether the observed differences we observed *in vitro* between wild-type BC7 and the BC7 *cblA,* BC7 *cblS*, and BC7 *adhA* mutants held true *in vivo*. In our previous studies, we showed that suspension of bacteria in *P. aeruginosa* alginate facilitates BC7 persistence in both normal and CFTR knockout mice, but the latter develop severe pneumonic consolidation in the lungs with 30% of the mice dying by 5 days [12]. This high mortality rate makes it difficult to follow bacterial persistence throughout the course of infection, therefore, we used normal mice to determine the capacity of the BC7 *cbl* and BC7 *adhA* mutants to persist and cause lung inflammation *in vivo*. To initiate these studies, we characterized the *B. cenocepacia* infection model with the wild-type strain BC7 in C57BL/6 mice with regards to bacterial persistence and related lung inflammation. We infected C57BL/6 mice with BC7 suspended in PBS (BC7/PBS) or alginate (BC7/alginate) by the intratracheal route. Mice were sacrificed at 1 h, 6 h, 1, 3 or 5 days post-infection, and examined for lung bacterial load and inflammation. Mice, mock-infected with PBS or alginate alone served as controls. As expected, these mock-infeccted mice did not show bacteria in their lungs. Mice infected with BC7/PBS showed abundant bacteria in their lungs up to 24 h after infection (Figure 2A), which was similar to the bacterial load observed in mice infected with BC7/alginate (Figure 2B). While all mice in the BC7/PBS group cleared bacteria by 3 days after infection, animals in the BC7/alginate group showed bacteria in their lungs up to 5 days, though there was a gradual decrease in this number. The difference between the BC7/PBS and the BC7/alginate groups was not due to initial deposition of bacteria, because both groups of mice showed a similar bacterial load 1 h post-infection as well as 1 day post-infection. These results suggest that alginate facilitates persistence of BC7 in normal mice similar to that observed in CFTR knockout mice [12]. However, unlike CFTR knockout mice, these infected C57BL/6 mice showed no mortality. Consistent with this, alginate has also been shown to increase the persistence of *P. aeruginosa* in BALB/c mice up to 7 days with no mortality [14].

**Figure 3:**
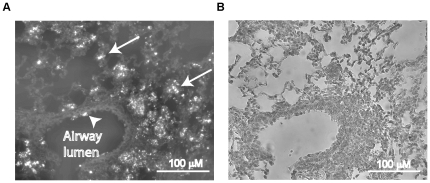
Immunolocalization of *B. cenocepacia* in mice infected with BC7/alginate. Mice were infected with BC7/alginate and sacrificed one day after infection. Lungs were fixed and embedded in paraffin. Lung sections were deparaffinized and incubated with antibody to *B. cenocepacia*. Bound antibody was detected by anti-rabbit IgG conjugated with Alexafluor 598 and sections were visualized under fluorescence microscope. A, lung section showing bacteria in both conducting (arrowhead) and respiratory zone (arrow). B, bright field image of A. Images are representative of two independent experiments.

To obtain persistent infection with *B. cenocepacia*, other investigators have adapted the agarose bead model that was originally developed for *P. aeruginosa* infection by Cash et al. [19], [20], [21]. Although this method is valuable in establishing chronic infection, it often facilitates infection of conducting airways, likely due to mechanical blocking of bronchioles by the beads [22]. In addition, this damage may mask the inflammation caused by bacteria [22]. In contrast, we and others have shown that the alginate suspension alone does not cause lung inflammation in mice [12]. Further, the mechanical entrapment of bacteria in beads may prevent spreading of bacteria to respiratory zone. This led us to examine the location of bacteria in the lungs of mice infected with BC7 suspended in alginate by immunolocalization. Lung sections immunostained with antibody to the bacteria showed BC7 in the airway lumen as well as in the respiratory zone associated with alveolar septa and inflammatory cells in these mice, suggesting that alginate does not restrict bacteria to conducting airways (Figure 3A and 3B). This distribution was very similar to that observed in CFTR knockout mice infected with *B. cenocepacia* suspended in alginate [12] as well as that observed in CF patients colonized with *B. cenocepacia* [23].

### Bacteria suspended in alginate stimulate altered cytokine responses in C57BL/6 mice

**Figure 4:**
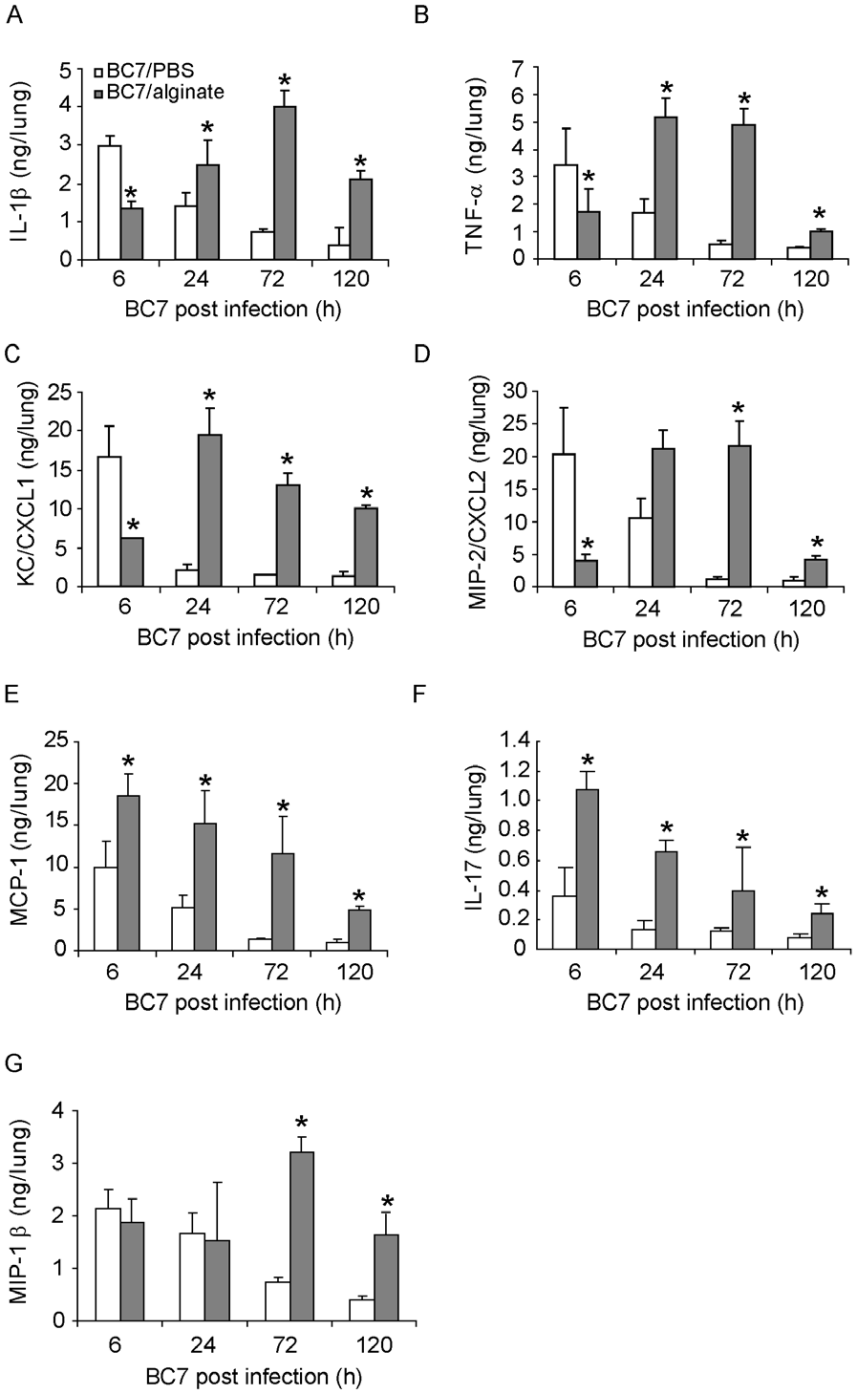
Cytokine levels in lungs of mice infected with BC7. Mice infected with BC7/PBS or BC7/alginate were sacrificed at specified times, and the cytokine levels in lung homogenates were measured by Bio-Plex multiplex immunoassay. A, IL-1β; B, TNF-α; C, KC/CXCL1; D, MIP-2/CXCL2; E, MCP-1; F, IL-17; and G, MIP-1β. Data represents mean and SEM calculated from three independent experiments with a total of 6 - 7 mice per group (*different from BC7/PBS group at respective time points, p≤0.05, ANOVA).

Chemokines and cytokines play an important role in recruiting phagocytes to the site of infection and subsequent clearance of bacteria. To examine whether alginate increases persistence of bacteria by delaying the initial chemokine and cytokine responses in normal mice, we measured the levels of selected chemokines and cytokines in lung homogenates obtained from uninfected control mice, and mice infected with BC7/PBS or BC7/alginate. Mice mock-infected with PBS or alginate alone showed very low or undetectable levels of all the measured cytokines and chemokines at all the time points tested (data not shown). In contrast, mice infected with either BC7/PBS or BC7/alginate showed a significant increase of all cytokines measured compared to the respective sham-infected groups, but with different kinetics (Figure 4A-G). Mice infected with BC7/PBS showed peak induction of KC/CXCL1, MIP-2/CXCL2, IL-1β, TNF-α, as early as 6 h, correlating with the bacterial load in the lung. The levels of these chemokines and cytokines gradually decreased and returned to normal by 3 d post infection. In contrast, mice infected with BC7/alginate showed increased KC/CXCL1, MIP-2/CXCL2, IL-1β and TNF-α at 6 h post infection; these levels were significantly lower than those observed in mice infected with BC7/PBS, despite similar bacterial load. The levels of these cytokines and chemokines in the BC7/alginate group peaked at 24 h post infection and was sustained up to 5 days, although the levels of these chemokines and cytokines decreased by day 5. We observed similar delayed chemokine responses in CFTR knockout mice infected with BC7/alginate, but in these mice the chemokine levels were sustained up to 7 days post infection [12].

KC/CXCL1 and MIP-2/CXCL2 are potent chemoattractants for phagocytes, and IL-1β and TNF-α, which stimulate the expression of KC/CXCL1 and MIP-2/CXCL2, play a critical role in recruitment of phagocytes to the site of infection and subsequent clearance of bacteria. Therefore, the delay in chemokine response as observed in the BC7/alginate group in normal mice may result in delayed phagocyte recruitment and attenuated bacterial clearance, and thus promote the establishment of bacterial infection. Consistent with our hypothesis, DBA2 mice, which is a susceptible mouse strain, are deficient in bacterial clearance due to an initial delay in phagocyte recruitment [24].

Compared to sham-infected mice, animals infected with either BC7/PBS or BC7/alginate showed significant increases in MCP-1, which recruits monocytes, and IL-17, a cytokine expressed by a subset of CD4 positive T cells. However, at all the time points examined, both MCP-1 and IL-17 were much higher in the BC7/alginate group compared to the BC7/PBS group. In addition, while the levels of MCP-1 and IL-17 returned to almost baseline in the BC7/PBS group by 3 days, mice in the BC7/alginate showed significantly increased levels of these cytokines up to 5 days, compared to sham-infected animals. The level of IL-17 has been shown to be increased in CF airways and is implicated in the increased chemokine responses and development of tissue inflammation [25], [26], [27]. Therefore, it is conceivable that sustained increased levels of IL-17 observed in the BC7/alginate group may contribute to increased chemokine response observed during the late phase of infection (1 to 5 days post infection). We also observed increased levels of MIP-1β/CCL4 at 3 days post infection in the BC7/alginate group compared to the BC7/PBS group. Since MIP-1β also increases the production of IL-1β and TNF-α from leukocytes, it may also contribute to an increased chemokine and cytokine response during the later stage of infection in the BC7/alginate group. Together these results suggest that alginate may facilitate persistence of bacteria by delaying the initial chemokine and cytokine response that is required for recruitment of phagocytes. The increased levels of chemokines that is observed in the BC7/alginate group may be due to both the persistent bacterial load and also exaggerated chemokine response stimulated by the increase in IL-17 and MIP-1β.

**Figure 5:**
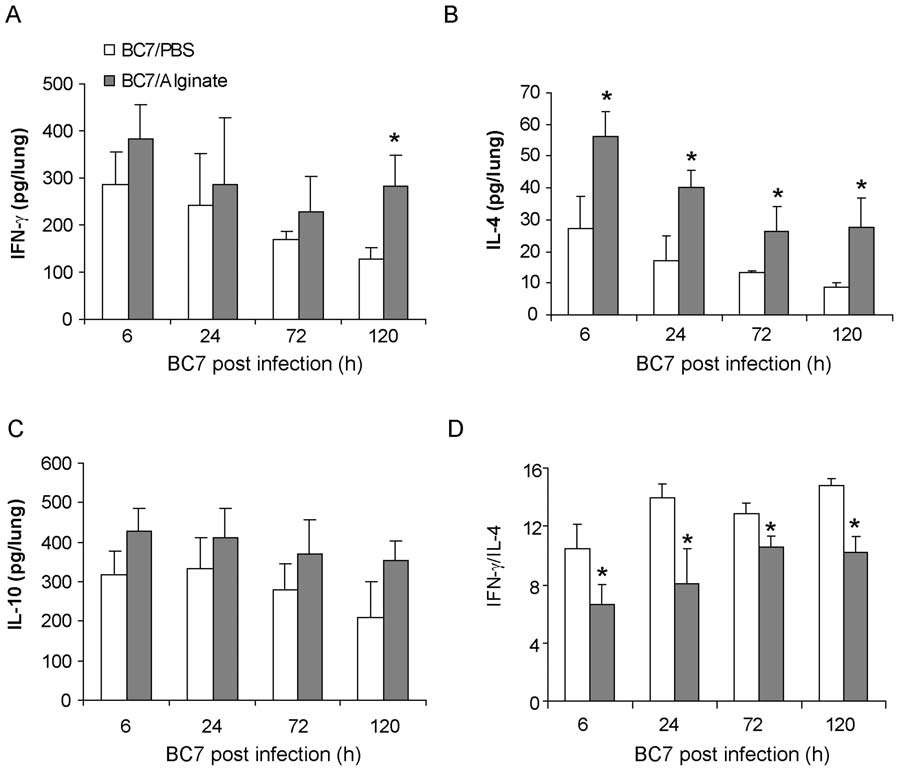
Th1 and Th2 cytokine levels in mice infected with BC7. Mice infected with BC7/PBS or BC7/alginate were sacrificed at specified times and levels of IFN-γ (A), IL-4 (B), and IL-10 (C) in lung homogenates were measured by Bio-Plex multiple immunassay. D, ratio of IFN-γ to IL-4. Data represents mean and SEM calculated from three independent experiments with a total of 6 - 7 mice per group (*different from BC7/PBS group at respective time points, p≤ANOVA).

Th1 response following bacterial infection has been shown to be beneficial to the host. CF patients, who are chronically infected with *P. aeruginosa*, show predominantly a Th2 response that is correlated with a poor prognosis [28]. *P. aeruginosa* alginate was demonstrated to be refractory to a Th1 immune response [29]. To examine whether alginate protects *B. cenocepacia* by a similar mechanism, we examined the levels of IFN-γ, IL-4, and IL-10, after infection. There was no significant difference in the levels of IFN-γ (which is Th1 cytokine) and IL-10 (which suppresses expression of pro-inflammatory cytokines) between the BC7/PBS and BC7/alginate groups up to 3 days post-infection (Figure 5). Interestingly, at 5 days post-infection, the BC7/alginate group showed significantly higher levels of IFN-γ than the BC7/PBS group. IL-4 (which is a Th2 cytokine) was also significantly increased in the BC7/alginate group at all the time points compared to the BC7/PBS group. The ratio between IFN-γ to IL-4 is used as an indicator of Th1 versus Th2 responses. Compared to the BC7/PBS group, the BC7/alginate group showed a significantly decreased IFN-γ/IL-4 ratio, suggesting BC7 may tip the balance towards Th2 type of response in the presence of alginate and that this may contribute to persistence of the bacteria.

### Neutrophil recruitment is delayed by alginate

**Figure 6:**
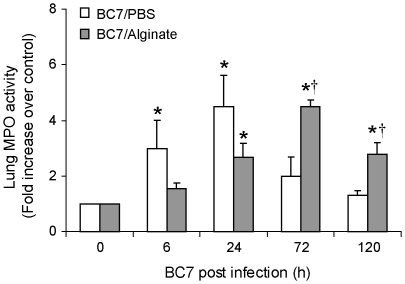
Lung MPO activity in BC7 infected C57BL/6 mice. Mice infected with BC7/PBS or BC7/alginate were sacrificed at specified times and MPO activity was determined in the lung homogenates. Data represents mean and SEM calculated from three independent experiments with a total of 6-7 mice per group (*different from respective uninfected group p≤0.05, †different from BC7/PBS group at respective time points p≤0.05, ANOVA).

Myeloperoxidase (MPO) is predominantly produced by activated neutrophils and can be used as a surrogate marker for their presence in the lung. Mice mock-infected with either PBS or alginate did not show MPO activity (data not shown). On the other hand, consistent with the cytokine levels, mice infected with BC7/PBS showed increased MPO activity as early as 6 h post-infection, with a peak activity at 24 h post-infection (Figure 6). MPO levels gradually decreased and returned to basal levels by 5 days in these mice. In contrast, mice infected with BC7/alginate did not show increased MPO activity 6 h post-infection, despite similar bacterial loads. This correlated with the attenuated chemokine and cytokine expression at 6 h post infection, observed in the BC7/alginate group. However, at 24 h post infection, the MPO activity increased in the BC7/alginate group reaching a maximum at 3 days corresponding to bacterial load and chemokine levels. Together, these results suggest that the initial delay in the recruitment of phagocytes to the site of infection in the BC7/alginate group prevents early clearance of bacteria thus providing suitable environment for bacteria to establish infection and spread to respiratory zone.

### Mice infected with BC7/alginate show persistent lung inflammation

**Figure 7:**
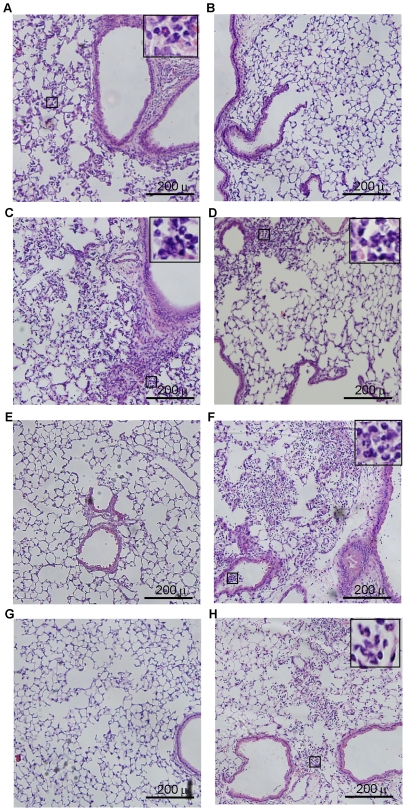
Lung inflammation in C57BL/6 mice infected with BC7/PBS or BC7/alginate. Paraffin lung sections from mice infected with BC7/PBS (A, C, E and G) or BC7/alginate (B, D, F and H) were stained with H&E. A and B, 6 h post-infection; C and D, 1 d post-infection; E and F, 3 d post-infection; and G and H, 5 d post-infection, respectively. Insets in A, C, D, F and H represent magnified view of areas marked in respective panels and show infiltrated neutrophils. Images are representative of 3 individual animals at each time point.

Next we examined the hematoxylin and eosin (H&E) stained lung sections to assess the inflammatory changes in mice infected with BC7/PBS or BC7/alginate. Neither PBS- nor alginate-treated mice showed lung inflammation at any of the time points examined (data not shown). Lung sections from mice infected with BC7/PBS showed mild inflammation at 6 h post-infection, with few neutrophils (Figure 7A). These mice showed moderate neutrophilic inflammation, particularly in peribronchiolar and perivascular areas at 1 d post-infection (Figure 7C), and mild and no inflammation, at 3 and 5 days post-infection, respectively (Figure 7E and 7G). In contrast, BC7/alginate-infected mice showed no inflammation at 6 h post-infection (Figure 7B) and mild neutrophilic inflammation at 24 h post infection (Figure 7D). However, at 3 d post-infection, mice infected with BC7/alginate showed severe widespread inflammation with neutrophil infiltration in both airway lumina and parenchyma (Figure 7F). At 5 d post-infection, these mice still showed inflammation (Figure 7H), but it was less compared to the mice at 3 d post-infection. Taken together these results suggest that alginate facilitates establishment of *B. cenocepacia* BC7 infection in normal mice similar to that observed in CFTR knockout mice. However, unlike CFTR knockout mice, normal mice show a trend towards clearing bacteria, resolving inflammation and also do not show mortality. Therefore, using this relatively well-characterized *B. cenocepacia* infection model in normal mice, we examined the capacity of BC7 *cbl* and BC7 *adhA* mutants to persist and cause inflammation *in vivo*.

### BC7 *cblA*, BC7 *cblS*, and BC7 *adhA* mutants are attenuated in their capacity to persist and cause lung inflammation in vivo

**Figure 8:**
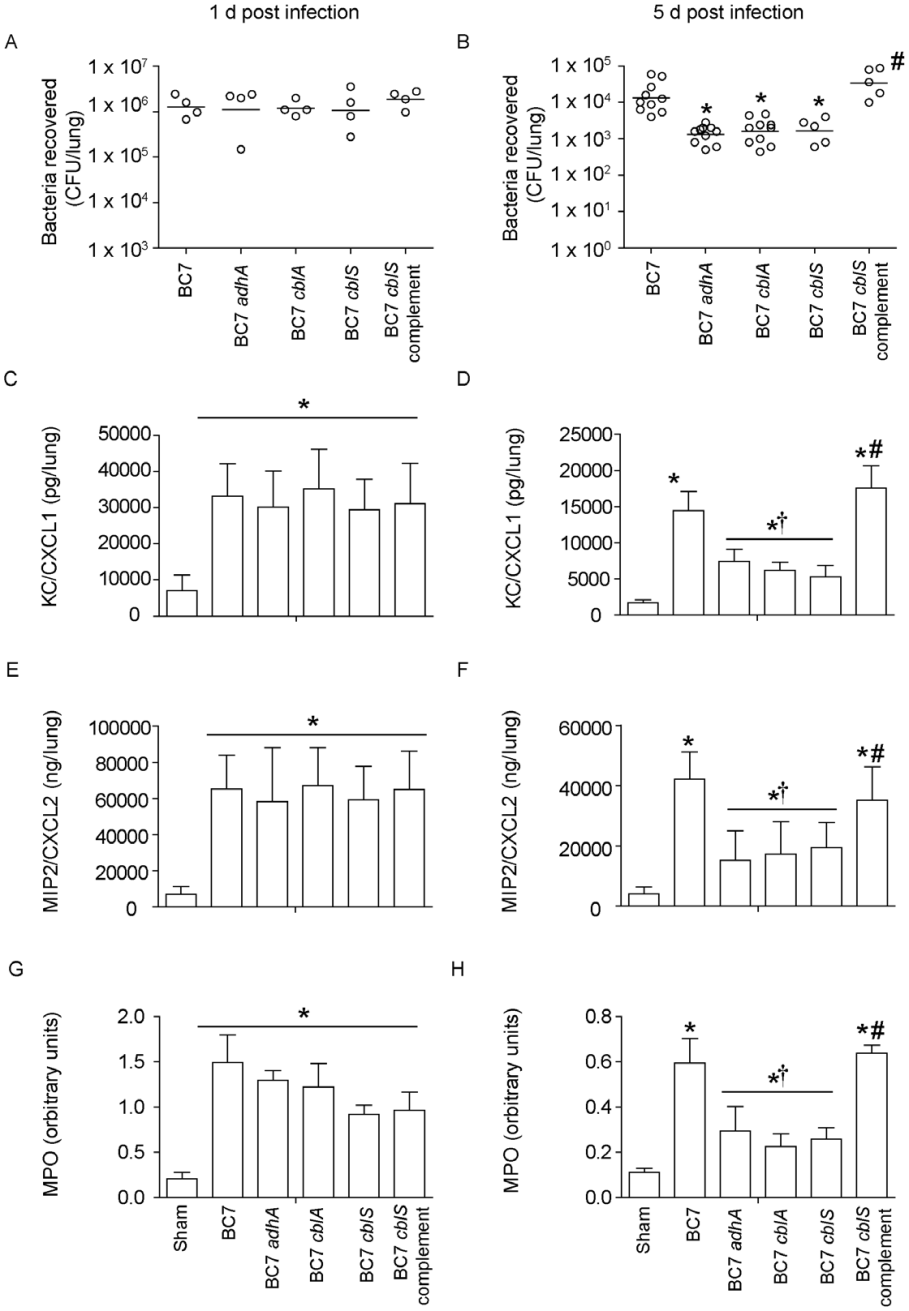
Bacterial load and chemokine responses in mice infected with *B. cenocepacia* strains. Mice were infected with wild-type BC7, the BC7 *adhA*, BC7 *cblA*, BC7 *cblS* mutants, or the BC7 *cblS* complement, suspended in alginate, and sacrificed at 1 or 5 d post-infection. Lung homogenates were used to determine bacterial load (A and B), KC (C and D), MIP-2 (E and F) and MPO activity (G and H). Data in represents either median with range (A) or mean with SEM (B–D) determined from two independent experiments with a total of 5 mice per group (*different from wild-type BC7, † different from BC7, #different from BC7 mutants p≤0.05, ANOVA on Ranks or ANOVA).

The C57BL/6 mice were infected with BC7 *cblA*, BC7 *cblS,* BC7 *adhA*, the BC7 *cblS* complemented strain or wild-type BC7; all bacteria were suspended in alginate. Mice were sacrificed 1 or 5 days after infection, and examined for lung bacterial load and levels of inflammatory markers. As observed earlier, mice infected with wild-type BC7 showed persistence of bacteria up to 5 days. On the other hand, mice infected with BC7 *cblA*, BC7 *cblS*, or BC7 *adhA* showed similar bacterial loads at 1 day post infection, but had significantly reduced bacterial loads at 5 day post infection, compared to mice infected with wild-type BC7 (Figure 8A). This is not surprising, because we have previously shown that during the initial phase of infection, alginate protects bacteria from phagocytes by altering bacterial surface properties and this in turn prevents rapid bacterial clearance from the lungs [12]. However at later stages of infection, only bacteria that have the capacity to colonize may persist and the others are cleared. Based on this notion, we believe that BC7 *cblA*, BC7 *cblS*, and BC7 *adhA* are attenuated in their capacity to colonize and persist in the lungs. The BC7 *cblS* complemented strain on the other hand, persisted in the lungs similar to wild-type BC7 indicating the contribution of cable pili in colonization and persistence. Collectively, these results suggest that both cable pili and adhesin contributes to BC7 persistence *in vivo*.

Next we measured the levels of KC (Figure 8C and 8D), MIP-2 (Figure 8E and 8F) , and MPO activity (Figure 8G and 8H) in mice infected with BC7, BC7 *cblA*, BC7 *cblS*, or BC7 *adhA*, or the BC7 *cblS* complemented strain, at 1 and 5 days post-infection. Both at 1 and 5 days post infection, mice infected with all strains showed increased levels of KC, MIP-2 and MPO compared to sham-infected animals. However, mice infected with wild-type BC7 showed significantly higher KC, MIP-2 and MPO levels than the mice infected with the BC7 *cblA*, BC7 *cblS*, or BC7 *adhA* at 5 days post infection, but not at 1 day post infection; and this may reflect the bacterial load in the lungs of these mice. Mice infected with the BC7 *cblS* complemented strain showed chemokine and MPO levels similar to wild-type BC7, indicating that the attenuated response observed with BC7 *cblS* was due to lack of cable pili expression. The observed decreased levels of chemokines and MPO activity levels in mice infected with the BC7 cable and adhesin mutants may be a combined effect of lower bacteria load and attenuated capacity of these strains to induce inflammation. Together, these results suggest that both cable pili and adhesin play a role in facilitating bacterial persistence and causing inflammation.

### BC7 *cblA*, BC7 *cblS*, and BC7 *adhA* mutants are attenuated in spreading infection to respiratory zone

**Figure 9:**
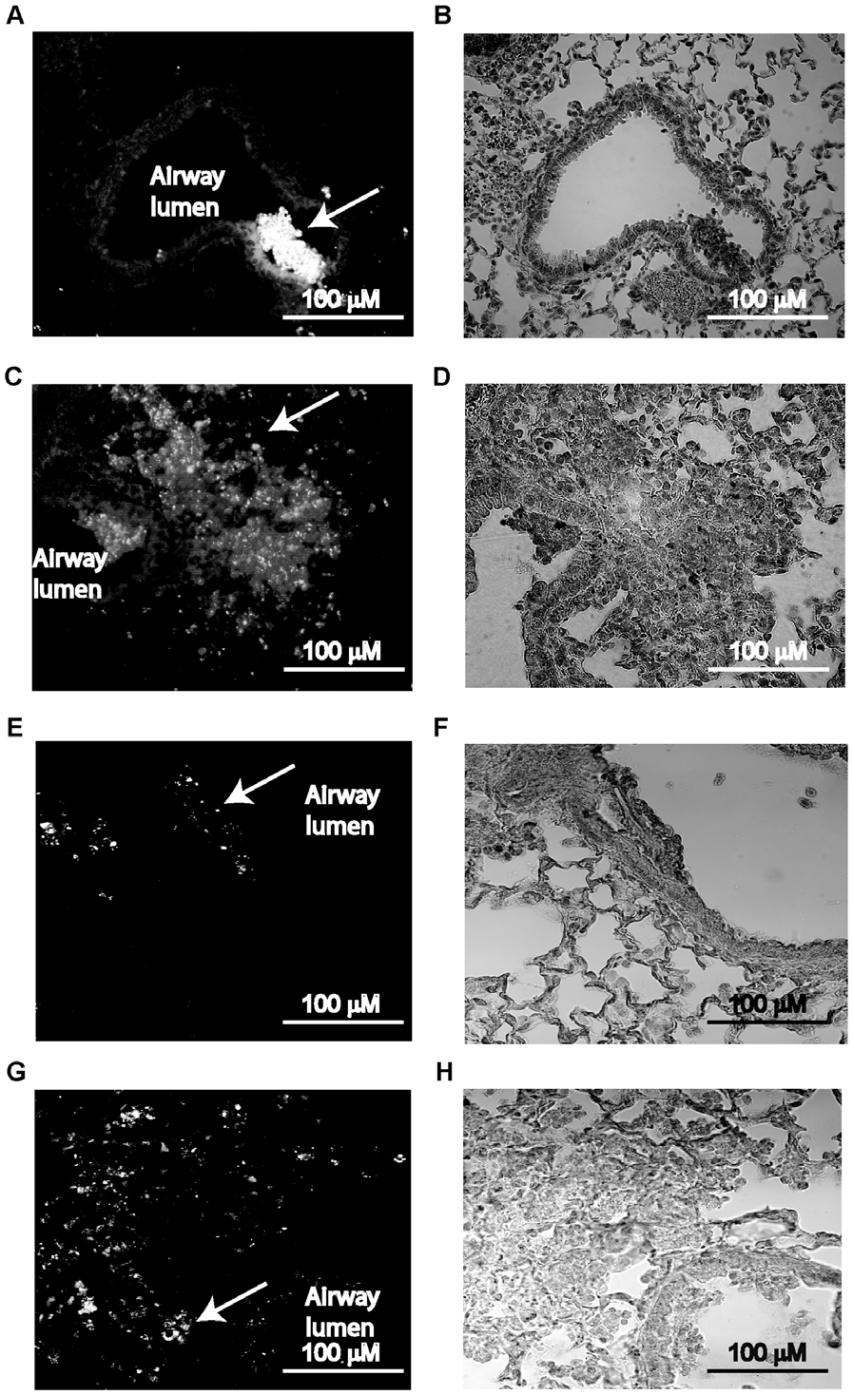
Immunolocalization of bacteria in mice infected with BC7 mutants or BC7 *cblS* complement. Mice were infected with the BC7 *adhA*, BC7 *cblA*, or BC7 *cblS* mutants or the BC7 *cblS* complement suspended in alginate and sacrificed at 1 d post-infection. Paraffin lung sections from these mice were deparaffinized and incubated with antibody to *B. cenocepacia*. Bound antibody was detected by second antibody conjugated with AlexaFluor 488 and sections were counterstained with hematoxylin. Sections were viewed under fluorescence microscope. A, C, E and G shows bacteria in lung sections from mice infected with BC7 *adhA*, BC7 *cblA*, BC7 *cblS*, and the BC7 *cblS* complement, respectively. B, D, F and H are bright field images of A, C, E and G respectively. Arrows point to bacteria in the lung. Images are representative of three mice per group.

To assess the capacity of the BC7 *cblA*, BC7 *cblS*, and BC7 *adhA* mutants to establish infection beyond the conducting airways, we determined the distribution of bacteria in lungs of mice infected with these mutants using antibody to *B. cenocepacia* [23]. In contrast to mice infected with wild-type BC7 (Figure 3B), mice infected with BC7 *adhA* (Figure 9A and 9B), BC7 *cblA* (Figure 9C and 9D), or BC7 *cblS* (Figure 9E and 9F), showed bacteria mostly in the airway lumen and in the peribronchiolar area, but not in the respiratory zone associating with alveolar septa. On the other hand, mice infected with the BC7 *cblS* complemented strain showed bacteria in both airways and in respiratory zone (Figure 9G and 9H) as was observed with wild-type BC7, suggesting that restoring cable pili expression restores capacity of BC7 *cblS* mutant to spread into peripheral part of the lungs. Previously we have shown that CF patients, who are colonized with *B. cenocepacia*, often show bacteria in parenchyma [23]. We and others have also shown that *B. cenocepacia* strains are capable of invading epithelium as well as transmigrating across polarized airway epithelial cells via the paracellular route [23], [30], [31], [32], [33]. Taken together, our results suggest that the spread of infection to respiratory zone may depend on bacterial capacity to invade airway epithelium and to subvert host innate immune mechanisms. These studies also suggest that both cable pili and adhesin are required at least in part to persist and cause inflammation *in vivo*.

### Conclusions

Our results demonstrated that alginate, a virulence factor produced by mucoid *P. aeruginosa*, prevents the initial clearance of *B. cenocepacia* by delaying the early innate immune defense mechanisms. This in turn facilitates persistence of those bacteria that possess the capacity to invade host mucosa and establish infection beyond the conducting airways. Further, we show that both cable pili and the associated 22 kDa adhesin protein are required for persistence and spreading the infection to respiratory zone. Both cable pili and the 22 kDa protein are also required for the stimulation of maximal IL-8 responses *in vitro* and inflammatory responses *in vivo*. Therefore, *B. cenocepacia* ET12 strains expressing both cable pili and the 22 kDa adhesin protein may cause persistent infection leading to severe inflammation in CF patients who are already colonized with *P. aeruginosa*.

## Materials and Methods

### Bacteria and growth conditions

*B. cenocepacia* isolate BC7, the BC7 *adhA*, BC7 *cblA*, and BC7 *cblS* mutants, and the BC7 *cblS* complemented strain have been described previously [11], [16]. All bacteria were maintained as glycerol stocks at −80°C and subcultured on brain heart infusion agar (BD Diagnostics, Franklin Lakes, NJ) and grown for 24 h at 37°C. BC7 mutant strains were cultured on Luria agar containing 1 mg/ml trimethoprim. The BC7 *cblS* complemented strain was grown overnight on Luria agar containing 1 mg/ml trimethoprim and 0.3 mg/ml tetracycline. A single colony was transferred to 10 ml tryptic soy broth (TSB, BD Diagnostics), TSB containing trimethoprim, or TSB containing trimethoprim and tetracycline, as appropriate, and grown overnight in a shaking incubator. Bacteria were harvested by centrifugation at 5,000×*g*, washed once with endotoxin-free PBS (Sigma-Aldrich, St. Louis, MO), and finally suspended in PBS or purified *Pseudomonas* alginate (prepared as described below) to the appropriate concentration based on OD_600_ (1 O.D. unit is equivalent to 1×10^9^ CFU/ml). The actual concentration of bacteria in a suspension was determined by plating.

### Cell cultures and infection

IB3 cells are immortalized CF airway epithelial cells and were obtained from Dr. P. Zeitlin (Johns Hopkins University School of Medicine, Baltimore) and were cultured in LHC-8 media (Invitrogen, Carlsbad, CA) containing 10% fetal bovine serum and L-glutamine. BEAS2B cells (American Type Culture Collection, Manassas, VA) are immortalized normal bronchial epithelial cells and cultured in bronchial epithelial cell growth media (Lonza, Walkersville, MD). For bacterial infection assays, cells were grown in 24-well cell culture dishes until 80% confluent. Cells were serum-starved overnight, infected with bacteria (at a multiplicity of infection (MOI)  = 1.0), and the media was collected at 6 h post-infection, for determination of cytokines by ELISA (R & D Systems, Minneapolis, MN).

### Isolation and purification of alginate

*P. aeruginosa* alginate was isolated as described previously from a clinical strain of mucoid *P. aeruginosa*, isolate NH57388A (kindly provided by Dr. N. Hoffmann (University of Copenhagen, Copenhagen, Denmark)) [12], [14], [15]. For infection of mice, bacteria were suspended in 1.8 mg/ml alginate and a 50 µl of this suspension (90 µg of alginate per mouse) was used.

### Infection of animals

Ten to twelve week-old C57BL/6 mice were purchased from Charles River (Wilmington, MA) and maintained in a specific pathogen-free barrier facility in micro-isolator cages throughout the experiment. Mice were acclimatized for two weeks before infection. All the procedures performed on the mice were approved by the Animal Care and Use Committee of the University of Michigan (Approval Identification number 09527). Mice were anesthetized by intraperitoneal injection of xylazine (1 mg/kg body weight) and ketamine (50 mg/kg body weight) and infected with 50 µl of PBS, alginate (sham), or bacteria (5×10^6^ CFU) suspended in either PBS (BC7/PBS) or alginate (BC7/alginate) by the intratracheal route, as described previously [14], [15]. For experiments with other strains, bacteria were suspended in alginate. Mice were sacrificed at predetermined times, as indicated, by intraperitoneal injection of 0.3 ml of 30% pentobarbital.

### Lung bacterial load

Lungs were harvested aseptically and homogenized in sterile PBS. Ten-fold serial dilutions of lung homogenates were plated on *B. cepacia* isolation agar (BCSA) [34].

### Analysis of lung cytokines and myeloperoxidase activity

Lung homogenates were prepared in the presence of protease inhibitors, centrifuged and supernatants were used in cytokine analysis by Bio-Plex multiplex immunoassay (Biorad, Hercules, CA). Lung myeloperoxidase (MPO) activity was quantified, as described previously [35].

### Histopathology and immunolocalization of bacteria

Lungs were inflation fixed via the trachea with 10% formalin overnight and embedded in paraffin. Lung sections (5 µm thick) were stained with hematoxylin and eosin (H&E). Immunolocalization of bacteria in the lung sections was performed as described previously using antibody to *B. cenocepacia*, R418 [23].

### Statistical Analysis

Results are expressed as mean ± SEM or a range of data with geometric mean. Data were analyzed by using SigmaStat statistical software (Systat Software, Inc., San Jose, CA). To compare groups, one-way analysis of variance (ANOVA) with Student's t test or Tukey-Kramer post-hoc analysis, or ANOVA on Ranks with Dunn's post-hoc analysis was performed, as appropriate. A ‘p’ value ≤0.05 was considered significant.
